# Incorporation of Hydroxyethylcellulose-Functionalized Halloysite as a Means of Decreasing the Thermal Conductivity of Oilwell Cement

**DOI:** 10.1038/s41598-018-34283-0

**Published:** 2018-11-01

**Authors:** Junsang Cho, Gregory R. Waetzig, Malsha Udayakantha, Claire Y. Hong, Sarbajit Banerjee

**Affiliations:** 1 Department of Chemistry, Texas A&M University, College Station, TX 77843-3255 USA; 2 Department of Materials Science & Engineering, Texas A&M University, College Station, TX 77843 USA; 3 Cenovus Energy, Inc., 500 Centre St. S., Calgary, AB T2P 0M5 Canada

## Abstract

The significant heat loss and severe thermal fluctuations inherent in steam-assisted gravity drainage (SAGD) and cyclic steam stimulation (CSS) impose considerable constraints on well cementing. In order to obtain better energy efficiency and mechanical robustness, there is considerable interest in the development of low-thermal-conductivity cement that can provide a combination of enhanced thermal insulation and mechanical resilience upon thermal cycling. However, the current palette of thermal cements is exceedingly sparse. In this article, we illustrate a method for decreasing the thermal conductivity of cement by inclusion of hydroxyethylcellulose-functionalized halloysite nanotubes. Halloysite/hydroxyethylcellulose inclusions offer an abundance of disparate interfaces and void space that can effectively scatter phonons, thereby bringing about a pronounced reduction of thermal conductivity. The microstructure of the nanocomposite cementitious matrix is strongly modified even as the compositional profile remains essentially unaltered. Modified cement nanocomposites incorporating halloysite nanotubes along with hydroxyethylcellulose in a 8:1 ratio with an overall loading of 2 wt.% exhibit the lowest measured thermal conductivity of 0.212 ± 0.003 W/m.K, which is substantially reduced from the thermal conductivity of unmodified cement (1.252 W/m.K). The ability to substantially decrease thermal conductivity without deleterious modification of mechanical properties through alteration of microstructure, inclusion of encapsulated void spaces, and introduction of multiple phonon-scattering interfaces suggests an entirely new approach to oilwell cementing based on the design of tailored nanocomposites.

## Introduction

Well cementing, the pumping and solidification of cement within the annulus between the inserted casing and its surrounding rock formation, represents a critically important step in the completion of oil wells^1^. The oilwell cement provides zonal isolation, protects surrounding groundwater from contamination, structurally bolsters the casing, and substantially mitigates corrosion of the casing. The choice of cement and the method of cementing are determined by the specifics of the geological formation and the nature of the extraction process. Enhanced oil recovery methods that are increasingly being used to meet global energy needs often have specific requirements for well cementing^2^, which adds considerable cost and complexity to the myriad challenges of accessing unconventional deposits. In recent times, the steam-assisted gravity drainage (SAGD) process has emerged as one of the pre-eminent means of extracting viscous oil in the Canadian Oil Sands spread across Northern Alberta and Saskatchewan^3,4^. In this process, steam is injected down a wellbore through an injection well creating a steam chamber that increases the temperature of the viscous oil deposits, thereby reducing the viscosity of the oil and allowing it to drain down to a lower production well (under the influence of gravity) from which it is extracted using powerful pumps as an emulsified mixture of water and oil^5,6^. Apart from load-bearing characteristics, the cement components deployed in SAGD wellbores also have to endure the severe thermal cycling inherent in the SAGD process; the oilwell cement must further be flexible and yet dense in order to avoid microannulus formation, adhere conformally to metal tubing, and provide zonal isolation^7–9^. Given the considerable temperature differential inevitable between the casing and the environment during the SAGD process, there is substantial interest in reducing the thermal conductivity of the oilwell cement in order to reduce heat loss and improve the energy efficiency of the SAGD process. Needless to say, a reduction in thermal conductivity needs to be accomplished without deleteriously impacting critically important mechanical properties. In this article, we report a pronounced reduction in the thermal conductivity of oilwell cement upon inclusion of polymer-functionalized halloysite nanotubes (HNTs). The hollow interiors of the HNTs and the scattering of phonons at multiple interfaces yields cement with a substantially decreased thermal conductivity without degrading the mechanical properties of the cement.

Concrete cement is a mixture of silicates and oxides of calcium, aluminum, and iron, primarily comprising various phases of calcium and aluminum silicates. Upon the addition of water, hydraulic cement can be quickly gelled through the hydration reaction of various metal silicates^10–13^. Several thermal cements incorporating polymeric and polysulfide additives have been developed in order to provide enhanced elastic deformability and thermal insulation^14^. Increasing the flexibility (by decreasing the Young’s modulus) of the cementitious matrix helps prevent delamination at the metal tubing interface and further prevents buildup of strain gradients (derived in large measure from thermal gradients) that could bring about fracture and compromise well integrity^14^. Polymer-derived cements incorporate cross-linkable polymeric chains that are oftentimes covalently bonded to the cement matrix giving rise to a dense load-bearing framework. The ability to unravel polymeric chains and the flexibility of polymeric backbones can substantially enhance the elastic properties of the matrix. As a result of their “softer” more deformable characteristics, the Young’s modulus is decreased for such materials, albeit oftentimes with an accompanying reduction of compressive strength. Polymer-derived cements further serve to reduce water sorption, allow for enhanced corrosion resistance, and provide a means for improved adhesion to metal tubing. Polymeric inclusions further bring about some diminution of thermal conductivity as a result of the dissimilar nature of polymers and silicates, which results in considerable phonon scattering at their interfaces, thereby diminishing the thermal conductivity of the framework. Considerable efforts have also been directed at the inclusion of fibrous additives such as carbon nanotubes (CNTs) or plant fibers within cementitious matrices as a means of simultaneously enhancing deformability as well as fracture toughness^8,14–16^. A major challenge in the preparation of cement nanocomposites, which is imperative to fully harness the functional benefits of the inclusions, is to ensure homogeneous dispersion of the inclusions within the cementitious matrix whilst precluding the entrainment of air.

While macroscopic porosity is most certainly deleterious to cement strength and well integrity, the incorporation of closed nanoscopic voids represents an intriguing means of embedding a low-thermal-conductivity medium. Naturally occurring hollow clay nanotubes provide a means of introducing nanoscopic voids if they can be incorporated within cementitious matrices in an appropriate manner but such an approach to modulate thermal conductivity has not hitherto been explored to the best of our knowledge. HNTs have a rather similar chemical composition (Al_2_Si_2_O_5_(OH)_4_) to cement but have not been extensively explored as cement additives^17–19^. Their porous tubular nature and deformability are promising for structural applications that require low thermal conductivity and high elastic modulus (estimated to be ca. 140 GPa). As a result of their high aspect ratios, they can effectively bridge multiple domains and thereby preclude crack formation even at low loadings of additives. In this work, we have contrasted the modulation of thermal conductivity induced upon inclusion of different fibrous additives, CNTs, HNTs, and jute fibers. Based on the superior modulation of thermal properties accessible using HNTs, we have further explored the evolution of the thermal conductivity and mechanical properties of nanocomposite cement as a function of the HNT/polymer loading within cement and the HNT:polymer ratio. An unprecedented decrease of thermal conductivity is engineered without deleterious modification of mechanical properties, suggesting an entirely new approach to oilwell cementing.

## Results and Discussion

As a result of their covalently bonded -M-O-M- (M = Ca, Al, Fe) as well as silicate frameworks, cementitious materials are typically characterized by a high compressive strength and a high Young’s modulus but have a relatively low tensile strength^20^. The incorporation of nanometer-sized inclusions provides a means of modulating properties of cement as a result of the structural reinforcement provided by harder or more elastic additives or as a result of the altered ratios of different crystalline silicates nucleated at the interfaces with the additives^21–23^. As with other hybrid nanocomposites, in order to fully realize the benefits of the nanoscopic filers, it is imperative to ensure optimal dispersion of the additives within the host matrix and to further ensure interfacial compatibility of the host matrix and the added fillers^24^.

**Figure 1:**
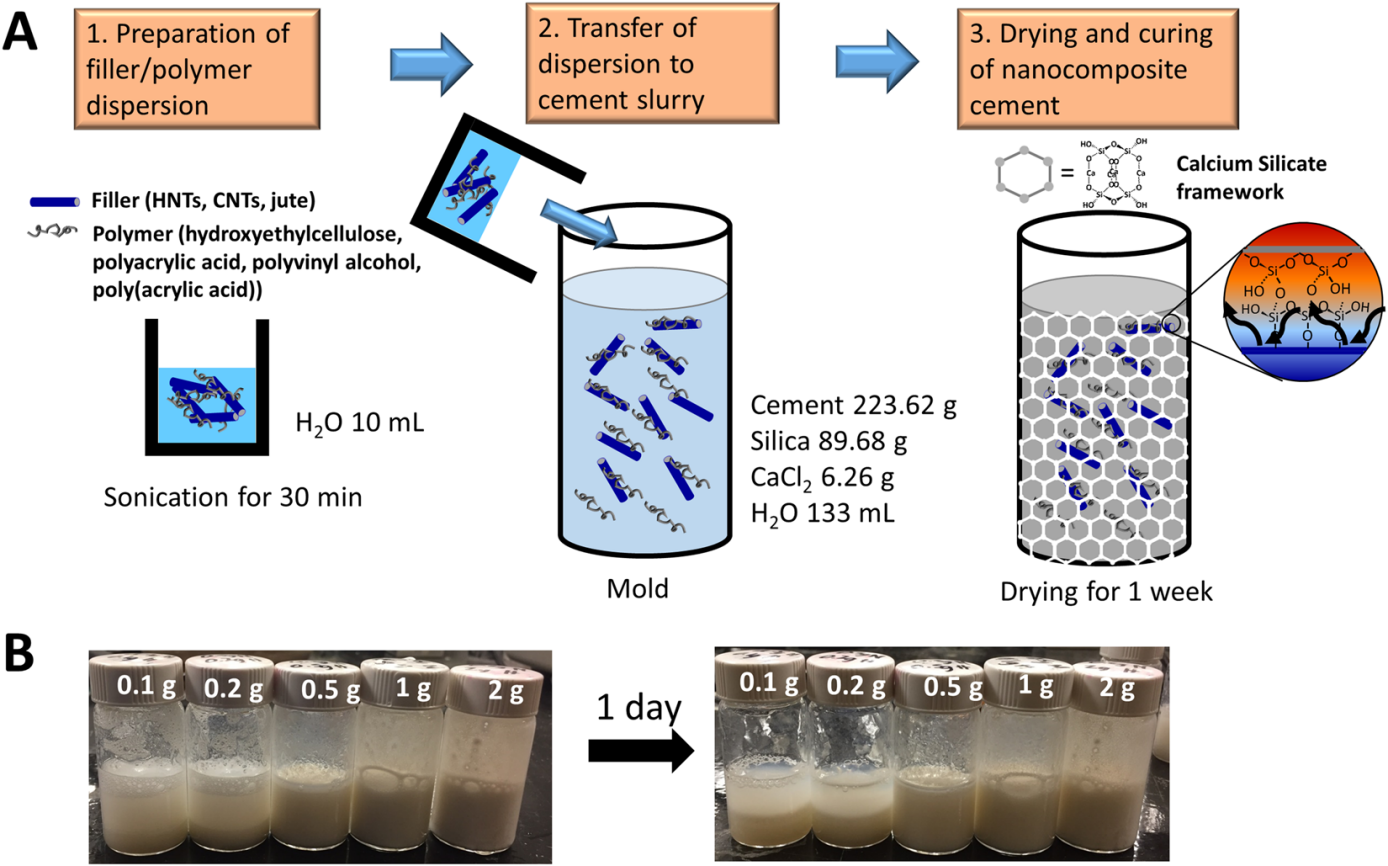
Schematic Illustration of the Preparation of Nanocomposite Thermal Cements Based on the Incorporation of Polymer-Functionalized Fillers within Cementitious Matrices. (**A**) Scheme for the design and preparation of the nanocomposite thermal cement incorporating polymer-modified fibrous fillers. An as-prepared dispersion of polymer-functionalized fibrous additives is added and vigorously mixed within a cement slurry, which is subsequently allowed to dry for 7 days resulting in solidification of the composite matrix. (**B**) Colloidal Dispersion of HNTs in water stabilized using hydroxyethylcellulose. Digital photographs of colloidal dispersions of HNTs dispersed in 10 mL water dispersed with the help of 0.5 g of hydroxylethylcellulose. The digital photographs in the left panel were taken immediately after mixing, whereas the panel on the right captures the dispersions 24 h after mixing.

Figure 1A presents a schematic depiction of the design and preparation of the nanocomposite thermal cement incorporating various polymer-encapsulated fibrous fillers. The initial step corresponds to blending of the fillers within the polymer matrix to obtain a dispersion of polymer-functionalized fillers. Polymers with polar functional groups are used to ensure aqueous dispersibility. Colloidal dispersions are obtained as a result of non-covalent binding of the polymers around the anisotropic fibrous additives^25^. The as-prepared dispersion is immediately transferred to the cement slurry and homogeneously mixed with the help of a mechanical stirrer to prepare a slurry incorporating the polymer-encapsulated fibers. The slurry is allowed to cure within a mold for 7 days to allow for cross-linking and stabilization of a rigid framework. The cement specimens prepared in this study include 2 wt.% of CaCl_2_, which reduces the setting and hardening time by enhancing the hydration rate, as is oftentimes the practice in field operations in the Canadian Oil Sands^26^.

As delineated in Fig. 1A, three distinct types of fillers have been incorporated within the nanocomposite cements, CNTs, HNTs, and cellulosic jute fibers. The relative ratio of the filler and the polymer and the overall loading of the filler within the cement matrix have been systematically varied. For the same mass of fillers used in the cement matrix, the dimensions, aspect ratio, and number density of additive fillers determine the extent of formation of percolative network (Supplementary Table 1). Notably, HNTs and CNTs have nanoscale dimensions, whereas jute fibers have macroscale dimensions. The aspect ratio of the fibers follows: HNTs < jute fibers < CNTs. The number density of fibers per unit volume of the cement matrix is deduced to be 3.7 × 10^12^ cm^−3^ for HNTs, 4.1 × 10^12^ cm^−3^ for CNTs, and 2.6 × 10^5^ cm^−3^ for jute fibers. The high number density and aspect ratio of CNTs readily facilitates establishment of a percolative network, the high number density but low aspect ratio of HNTs results in percolation only at high loadings, whereas the low number density and intermediate aspect ratio of jute fibers inhibits percolation but induces phase segregation. A preliminary screening of thermal conductivity has been performed for three different types of fillers and four distinct polymers at a fixed filler loading of 2 wt.% with a 4:1 weight ratio of filler/polymer (CNTs, HNTs, jute fibers as fillers; hydroxyethylcellulose, polyacrylic acid (Carbopol^TM^), poly(vinyl alcohol) (PVA), poly(acrylic acid) (Acrysol^TM^), as the polymers). Supplementary Table 2 summarizes the matrix of thermal conductivity values measured for different combinations of fibrous fillers and polymers.

The inclusion of polyacrylic acid within the cement matrix induces rapid gelling and segregation of the polymeric media from the cementitious matrix and furthermore is seen to be accompanied by considerable entrainment of air bubbles by stereomicroscopy (Supplementary Fig. 1). The presence of macroscopic voids is discernible and thus this polymer has not been further considered as an additive. The hydroxyethylcellulose nanocomposites showed considerably greater diminution of thermal conductivity for test specimens as compared to the polyacrylic acid and PVA blends (for all of the fibrous inclusions) and has thus been used as the polymeric dispersant in subsequent studies seeking to examine the evolution of thermal conductivity across a multidimensional compositional space.

Figure 1B illustrates digital photographs of colloidal dispersions of HNTs suspended in aqueous media using hydroxyethylcellulose as a dispersant at various particle loadings. The added amount of HNTs is varied from 0.1–2 g while keeping the mass of hydroxyethylcellulose constant at 0.5 g hydroxyethylcellulose in 10 mL water. Notably, even the highest loading of 2 g of HNT fillers shows excellent dispersion within the gelled hydroxyethylcellulose matrix 24 h after mixing. Similar stable dispersions are obtained for jute fibers and CNTs.

**Figure 2:**
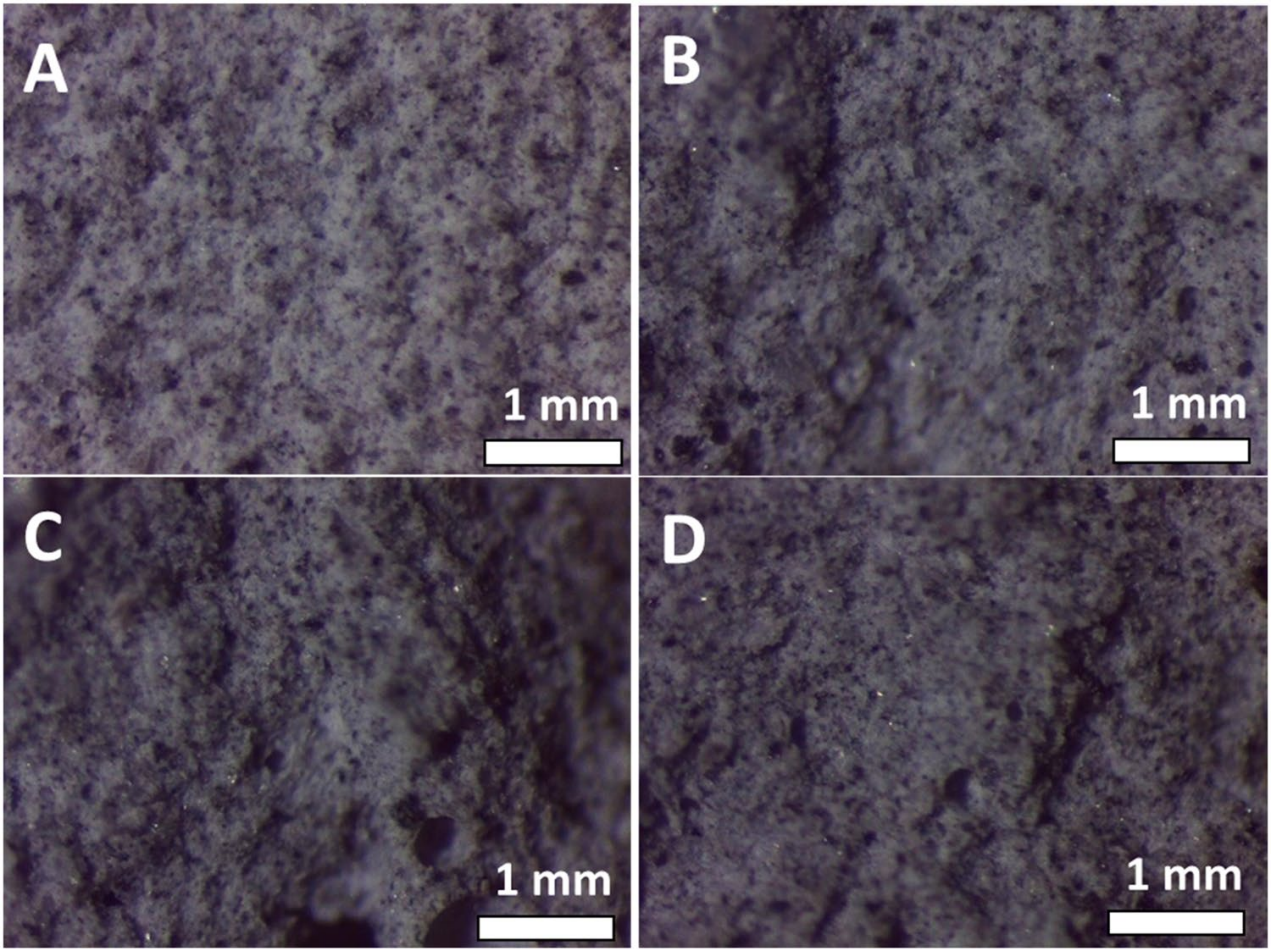
Stereomicroscopy Observations of Modified Cement Specimens. Stereomicroscopy images of (**A**) an unmodified cement specimen and modified cement nanocomposite incorporating 2 wt.% of polymer—fibrous additive inclusions: (**B**) HNTs dispersed with the aid of hydroxyethylcellulose; (**C**) jute fibers dispersed using hydroxyethylcellulose; and (**D**) CNTs dispersed using hydroxyethylcellulose. The fiber:hydroxyethylcellulose ratio is 4:1 in each case.

Figure 2 exhibits cross-sectional stereomicroscopy images of modified cement composites with 2 wt.% loading of various fillers (HNTs, CNTs, and jute fibers) functionalized with hydroxyethylcellulose; the unmodified cement specimen has also been imaged as a control. The unmodified cement shows a homogeneous distribution of particles at this magnification; similarly the modified cement nanocomposites are microscopically homogeneous suggesting the absence of phase segregation of filler particles from the host cement matrix; no evidence for microannuli (as observed for poly(acrylic acid) nanocomposites in Supplementary Fig. 1), which are severely detrimental to the cementing process^8^, is observed in any of the images. Microannulus formation has been strongly implicated in failures of well cementing in casing applications and thus it is notable that the fibrous inclusions at 2 wt.% loading do not give rise to microannuli detectable by stereomicroscopy^8,9^.

**Figure 3:**
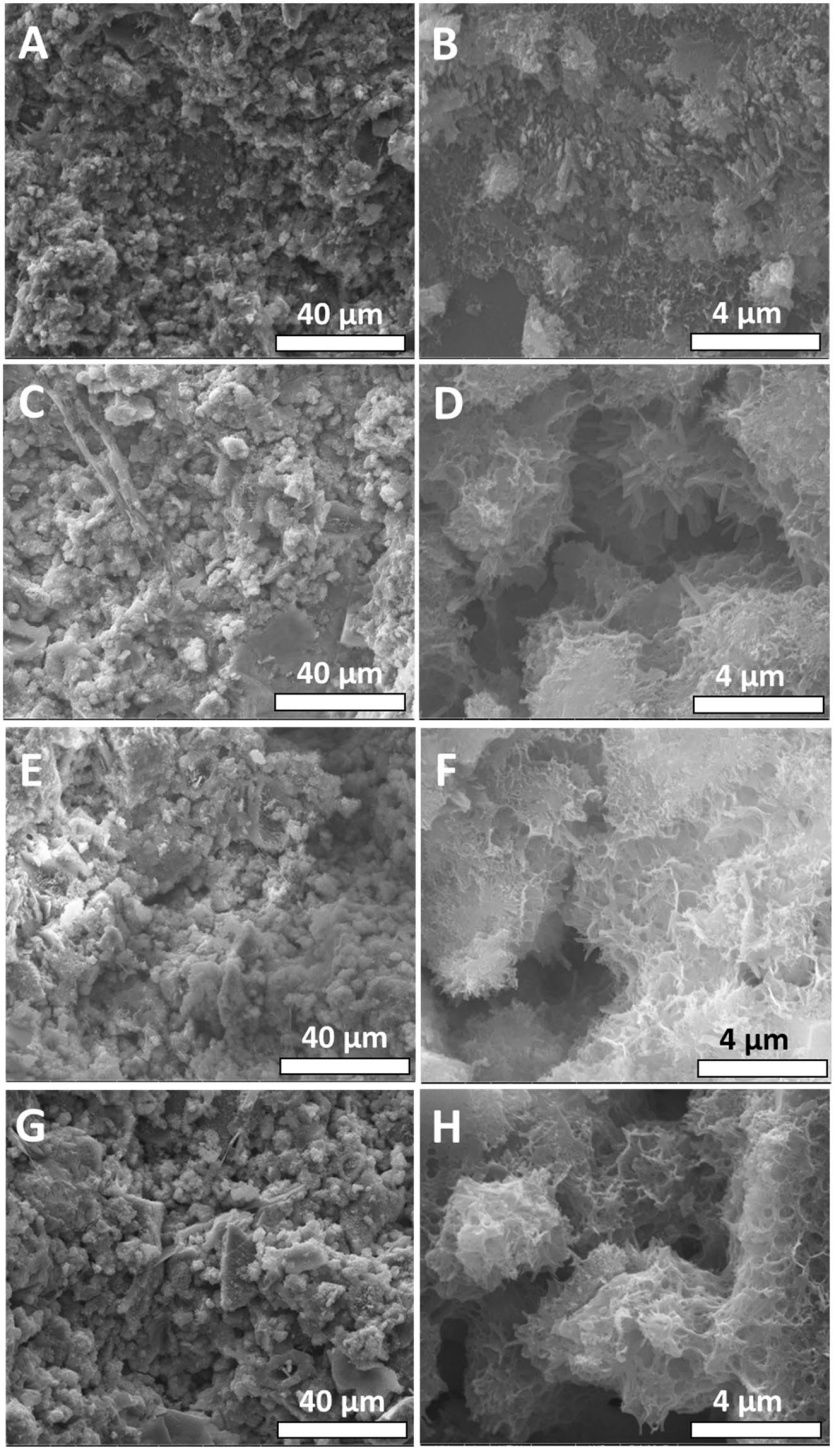
Microstructure of Cement Specimens with and without Fibrous Additives. SEM images of (**A**,**B**) unmodified cement; (**C**,**D**) cement nanocomposite incorporating a 2 wt.% loading of HNTs and hydroxyethylcellulose; (**E**,**F**) cement nanocomposite incorporating a 2 wt.% loading of jute fibers and hydroxyethylcellulose; and (**G**,**H**) cement nanocomposite incorporating a 2 wt.% loading of CNTs and hydroxyethylcellulose. The fiber:hydroxyethylcellulose ratio is 4:1 in each case.

The microstructures of the modified cement composites prepared using various fillers with hydroxyethylcellulose have been investigated by scanning electron microscopy (SEM) (Fig. 3). Low-magnification SEM images display the presence of aggregates of micron-sized primary particles. Similar micron-sized aggregates are observed in SEM images of the modified composites. High-resolution SEM images demonstrate that at the nanoscale level, the modified cement nanocomposites exhibit a distinctive hierarchical microstructure that is quite different from that of the unmodified cement specimen (Fig. 3B,D,F,H). A hierarchical porous microstructure is observed and is thought to derive from the shrinkage of the polymer-cross-linked cement particles upon drying (a similar microstructure is observed for all three fillers and thus appears to be primarily derived from the common hydroxyethylcellulose matrix). The stabilization of hierarchical nanoporous primary particles suggests an increased thermal contact resistance that has not been observed in unmodified cement (which in contrast exhibits a characteristic 3D interconnected microstructure), resulting in an increased resistance to phonon transport across macroscopic cement specimens in the former^27^.

**Figure 4:**
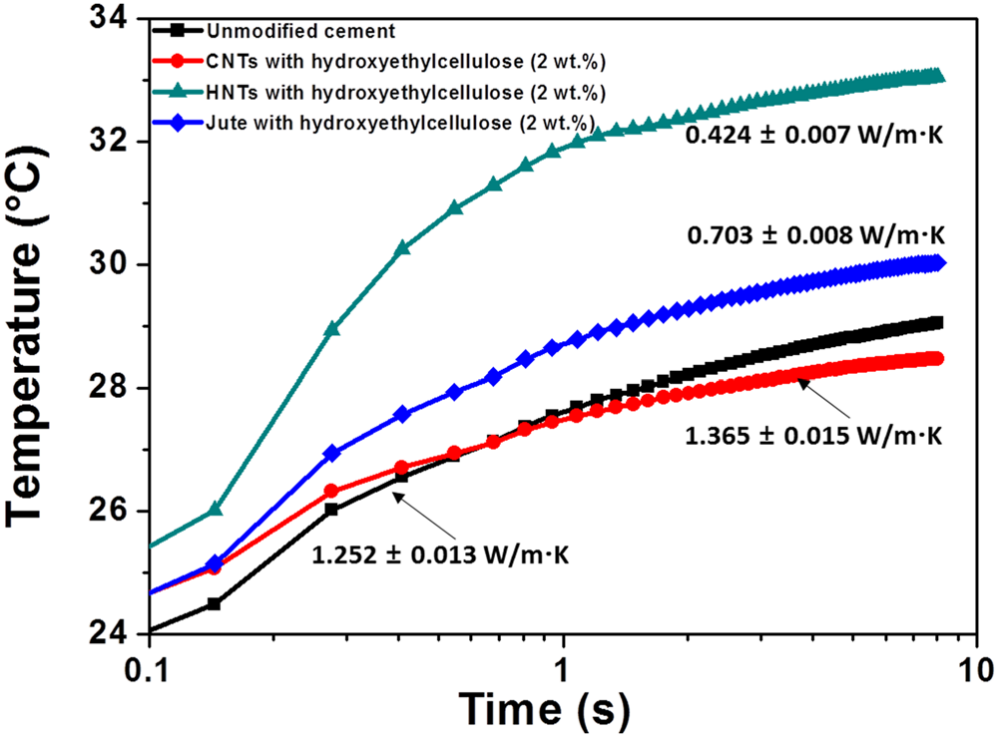
Thermal Conductivity of Cement Specimens with and without Fibrous Additives. Plots showing the evolution of temperature as a function of time for unmodified cement and modified cement composites containing 2 wt.% loadings of various fillers (HNTs, CNTs, and jute fibers) dispersed using hydroxyethylcellulose. The fiber:hydroxyethylcellulose ratio is 4:1 in each case.

Figure 4 contrasts the thermal conductivity profiles measured for unmodified and modified cement nanocomposites incorporating various filler/polymer additives (HNTs, CNTs, and jute fibers along with hydroxyethylcellulose) at a fixed loading of 2 wt.% using the transient hot bridge method. The deduced thermal conductivity values are 1.252 ± 0.013 W/m.K for unmodified cement; 0.424 ± 0.007 W/m.K for HNTs; 0.703 ± 0.008 W/m.K for jute fibers; and 1.365 ± 0.015 W/m.K for CNTs. The observed trend of thermal conductivity values is intriguing given the microstructure observed in Fig. 3. A hierarchically porous structure, derived from the use of the hydroxyethylcellulose, enhances the thermal contact resistance for all three types of fibrous additives, yet considerable differences are observed in the thermal conductivity values suggesting a pronounced dependence of thermal conductivity of the nanocomposites on the specific composition and structure of the fibrous fillers.

CNTs are excellent thermal conductors with thermal conductivity values approaching 3500 W/m·K along their length for individual single-walled carbon nanotubes^28^ and approaching 1500 W/m·K for aligned fibers^29^. Their high aspect ratios imply that they can readily form a quasi-percolative network across the 3D cement matrix (Supplementary Table 1), thereby yielding a higher thermal conductivity matrix despite the stabilization of the porous microstructure as a result of dispersion using hydroxyethylcellulose. In stark contrast, the diminution of thermal conductivity upon incorporation of HNTs is stark and can be attributed to the nanoscopic voids within such materials, which provides a low-thermal-conductivity medium. The inherent low thermal conductivity of HNTs (0.1 W/m.K) thereby brings about a pronounced decrease in the thermal conductivity of nanocomposites that include these materials. A decrease of thermal conductivity of phase-change materials when HNTs are used as storage media and an increase in thermal conductivity for carbon-based materials has been observed in a different context for phase change materials used for latent heat storage^30,31^. Jute fibers do not have the hollow voids characteristic of HNTs but being cellulosic in nature have low thermal conductivities (estimated to be 0.43 W/m·K)^32^ and also bring about a diminution of thermal conductivity of the cement specimens although not to the same extent as HNTs. In other words, the HNT/hydroxyethylcellulose inclusions offer an abundance of disparate interfaces and void space that can effectively scatter phonons, thereby reducing the thermal conductivity^17–19^. Indeed, the reduced thermal conductivity of composites incorporating hydroxyethylcellulose-modified HNTs is seen to hold with and without inclusion of 2 wt.% CaCl_2_ (Supplementary Fig. 2). Notably, the thermal conductivity of cement is increased with addition of CaCl_2_ as a result of reduction of porosity.

**Figure 5:**
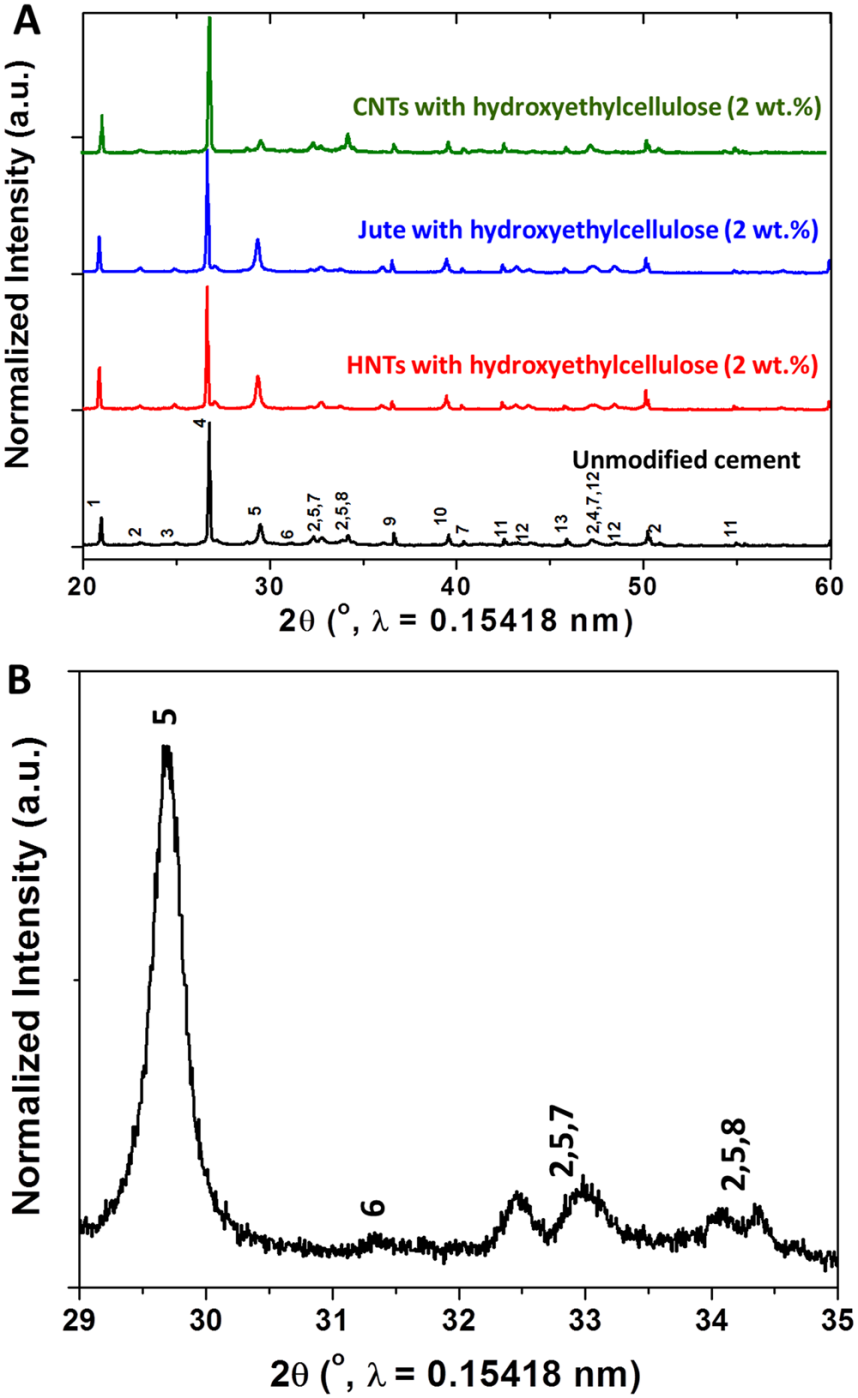
XRD Patterns of Cement with and without Inclusion of Hydroxyethylcellulose-Modified HNTs. (**A**) XRD patterns of unmodified cement (black) and modified cement nanocomposite incorporating 2 wt.% loadings of fibrous additives with hydroxyethylcellulose: HNT (red), Jute (blue), and CNT (green). (**B**) magnified XRD pattern of unmodified cement in the 2*θ* range between 29–35° confirming the presence of four major phases of cement in the unmodified cement specimen. The observed reflections are assigned to the following crystalline phases with the corresponding JCPDS number quoted in parenthesis in each case: 1. Ca_3_Al_2_O_6_ (32-0150); 2. γ-C_2_S (2CaO·SiO_2_; 31-0297); 3. Anhydrite (CaSO_4_; 37-1496); 4. CaCO_3_ (33-0268); 5. C_3_S (3CaO·SiO_2_; M1: 13-0272, M3: 42-0551); 6. α-C_2_S (2CaO·SiO_2_; 23-1042); 7. TCA (3CaO·Al_2_O_3_; 38-1429); 8. Ca_3_Al_2_O_6_ (32-0150); 9. Arcanite (05-0613); 10. Na_2x_Ca_3-x_Al_2_O_6_ (26-0957); 11. α-C_2_S (20-0237); 12. C_4_AF (4CaO·Al_2_O_3_·Fe_2_O_3_); 30-0226); 13. Sodium sulfate (Na_2_SO_4_; 37–1465,24–1132).

To investigate the crystallographic phases present within the cement composites, powder X-ray diffraction (XRD) patterns have been acquired for the modified cement composites at 2 wt.% loading of various fillers and are compared to the pattern of unmodified cement (Fig. 5A). The XRD pattern for unmodified cement (with the addition of CaCl_2_) verifies the presence of the major crystalline phases of Portland cement: (1) dicalcium silicate (belite; C_2_S; 2CaO·SiO_2_); (2) tricalcium silicate (alite; C_3_S; 3CaO·SiO_2_); (3) tricalcium aluminate (celite; C_3_A; 3CaO·Al_2_O_3_); and (4) tetracalcium aluminoferrate (ferrite; C_4_AF; 4CaO·Al_2_O_3_·Fe_2_O_3_)^11,33,34^. Figure 5B shows a magnified view of the powder XRD pattern acquired for unmodified cement indicating characteristic reflections of the C_3_S, C_2_S, and C_3_A phases in the 2*θ* range between 29–35°. The highest intensity reflection at a 2*θ* value of ca. 26° can be indexed to the (112) reflection of the vaterite phase of CaCO_3_ which is a major component of cement. It is worth noting that the powder XRD pattern remains essentially the same upon the inclusion and crosslinking of hydroxyethylcellulose-functionalized HNT, CNT, and jute fiber fillers. Indeed, this observation suggests that the crystallinity and compositional ratio of the cementitious matrix remains largely unaltered upon inclusion of 2 wt.% of the fillers even as the microstructure of individual particles is strongly modified. The diminution of thermal conductivity can thus be attributed to microstructural modifications and the specific role of the fillers themselves and not to a compositional or structural modification of the host matrix.

**Figure 6:**
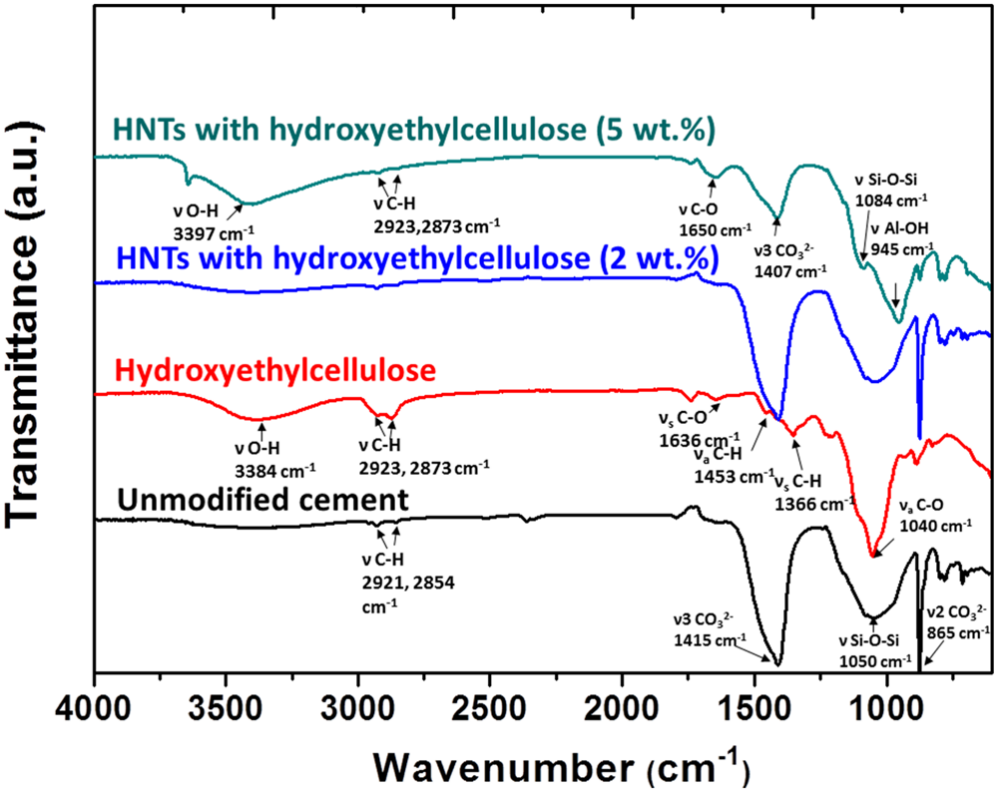
FTIR Spectra of Cement Specimens with and without Inclusion of Hydroxyethylcellulose-Modified HNTs. FTIR spectra measured for unmodified cement, hydroxyethylcellulose, and modified cement composites incorporating 2 and 5 wt.% loadings of hydroxyethylcellulose-modified HNTs.

Given the relatively higher diminution of thermal conductivity upon inclusion of HNTs, a detailed evaluation of HNT/hydroxyethylcellulose-modified cement has been undertaken at varying filler loadings. Figure 6 shows Fourier transform infrared (FTIR) spectroscopy data acquired for modified cement composites prepared with hydroxyethylcellulose-modified HNTs. The unmodified cement specimen displays prominent vibrational modes at 865, 1050, and 1415 cm^−1^, respectively, which can be assigned to ν_2_ CO_3_^2-^ stretching, Si-O-Si stretching, and ν_3_ CO_3_^2-^ stretching modes, respectively^35,36^; the modes arise from the CaCO_3_ and calcium silicate (C_3_S and C_2_S) phases that are the primary constituents of cement. Less intense modes are discernible at 2854^−1^ and 2921 cm^−1^ and can be assigned to CH_2_ stretching modes. The modified cement composites with 2 and 5 wt.% loading of HNTs added with hydroxyethylcellulose show intense IR bands centered around 1050–1100 cm^−1^ and 1400–1500 cm^−1^, which again can be assigned to Si-O-Si and CO_3_^2-^ stretching modes, respectively (as also observed for the unmodified cement specimen). The similarity in the vibrational spectra of the modified and unmodified cement specimens further attests to preservation of the primary phases. At a relatively high loading of 5 wt.% hydroxyethylcellulose-modified HNTs, a new vibrational mode at 945 cm^−1^ is observed and can be ascribed to the Al-OH stretch of the Al_2_Si_2_O_5_(OH)_4_ HNTs. A distinctive C—O stretching mode at 1650 cm^−1^ associated with elongation of the C-O vibration of cellulose rings is also observed for the modified cement specimens and derives from the incorporation of hydroxyethylcellulose within the cementitious matrix^37–39^.

**Figure 7:**
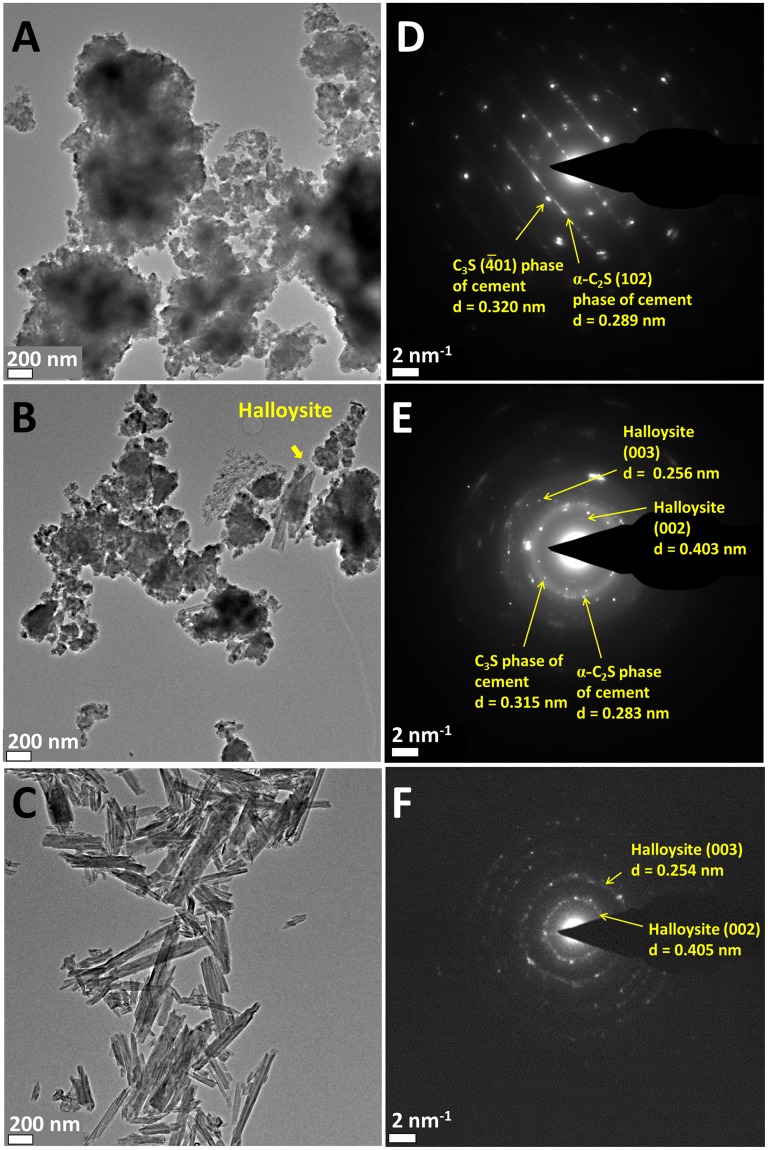
TEM Images of Cement with and without Inclusion of Hydroxyethylcellulose-Modified HNTs. TEM images of (**A**) unmodified cement (**B**) modified cement composite incorporating 2 wt.% of hydroxyethylcellulose-modified HNTs; and (**C**) HNT precursors. The corresponding SAED patterns are shown in (**D**–**F**); (**D**) the observed rings are indexed to the (−401) planes of C_3_S and (102) plane of C_2_S phase with d-spacings of 0.320 nm and 0.289 nm, respectively; (**E**) diffraction rings are indexed to C_3_S and C_2_S phases of cement and the (002) and (003) plane of HNTs; (**F**) diffraction rings are indexed to the (002) and (003) planes of HNTs with interplanar separations of 0.405 nm and 0.254 nm, respectively.

Figure 7 shows TEM images of unmodified cement and cement composites containing 2 wt.% of hydroxyethylcellulose-modified HNTs, contrasted with TEM images of the HNT precursors. Higher magnification images are shown in Supplementary Fig. 3. The HNTs are 40–50 nm in a diameter and span several micrometers in a length (Fig. 7C and Supplementary Fig. 3E,F). The hollow nature of the HNTs is clearly discernible in the TEM images. The TEM images directly evidence the inclusion of HNTs within the cement matrix and further establish that the HNTs retain their structural integrity and void space upon inclusion within the matrix. The unmodified cement sample depicts dense aggregates of the silicate and carbonate particles comprising stacked thin sheets and aggregated microparticles (Fig. 7A and Supplementary Fig. 3). The SAED pattern in Fig. 7B can be indexed to reflections from (−401) crystallographic planes of the C_3_S phase and (102) planes of α-C_2_S phase from unmodified cement^40^. Composite samples imaged after mechanical fracturing clearly show the presence of embedded HNTs (Fig. 7B and Supplementary Fig. 3C,D). Indeed, the high-aspect-ratio HNTs appear to connect primary particles in several instances, perhaps as a result of covalent tethering of the hydroxyethylcellulose to cement. The SAED pattern in Fig. 7D attests to the retention of crystallinity of the HNTs; the observed diffraction rings can be indexed to diffraction spots from (002) and (003) planes of HNTs along with reflections from the primary cement phases. These images thus provide direct evidence both of the nanoscopic voids and the multiple interfaces that result in the boundary scattering of phonons, thereby diminishing the thermal conductivity of cement.

The chemical homogeneity of cement samples before and after inclusion of hydroxyethylcellulose-modified HNTs has been further been mapped using energy dispersive X-ray spectroscopy (EDS). Supplementary Fig. 4 shows EDS spectra, whereas Supplementary Fig. 5 shows micron-scale elemental maps. Based on EDS mapping, the unmodified cement shows a homogenous distribution of Si (ca. 7.0 at.%), and Ca (ca. 10.4 at.%), O (ca. 54.1 at.%) across the entire area of the specimen, which suggests that the various calcium silicate phases are well-dispersed without compositional segregation or formation of large voids. Some minor contributions from other elements such as Al (ca. 0.4 at.%), S (ca. 0.6 at.%), K (0.4 at.%), Fe (ca. 0.3 at.%), Zr (ca. 0.6 at.%) are also identified. After inclusion of hydroxyethylcellulose-modified HNTs, the relative concentration of Si and Al is found to increase (Supplementary Fig. 4). However, EDS mapping suggests that the compositional homogeneity is maintained (Supplementary Fig. 5) confirming the homogeneous distribution of HNTs within the cementitious matrix as also discernible from the TEM images in Fig. 7.

**Figure 8:**
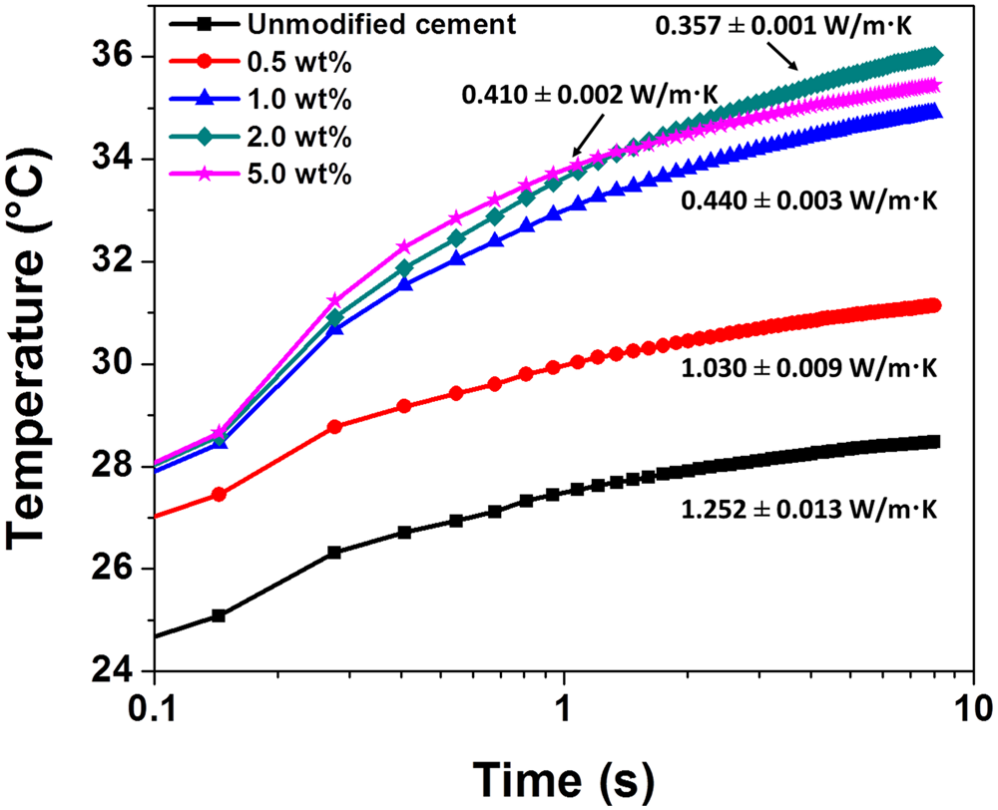
Thermal Conductivity of HNT-Modified Nanocomposite Cement Specimens as a Function of Filler Loading. Plots showing the evolution of temperature as a function of time measured using a hot bridge analyzer for unmodified cement and modified cement composites with varying loadings of HNTs. The HNT to hydroxyethylcellulose ratio is held constant at 4:1.

The HNT: polymer ratio and the overall loading of fillers have been systematically varied in the cement matrix. Figure 8 plots the temperature *versus* time profiles measured using a transient hot bridge analyzer for varying overall loadings of hydroxyethylcellulose-modified HNTs maintaining a constant polymer:HNT ratio of 4:1. The thermal conductivity values deduced from these measurements are 1.252 ± 0.013 W/m.K for unmodified cement, 1.030 ± 0.009 W/m.K for 0.5 wt.% hydroxyethylcellulose-HNT; 0.440 ± 0.003 W/m.K for 1.0 wt.% hydroxyethylcellulose-HNT; and 0.357 ± 0.001 W/m.K for 2.0 wt.% hydroxyethylcellulose-HNT. However, at 5.0 wt.% loading the thermal conductivity increases slightly to 0.410 ± 0.002 W/m.K. At relative high loadings of HNTs in the range of 2–5 wt.%, Supplementary Fig. 6 indicates that flakes of cement are bridged by HNTs. Such a bridging function can mitigate crack formation. However, at high loadings, it is likely that the low-aspect ratio HNTs begin to form a percolative network that makes available a new pathway for phonon transport along their crystalline walls (Supplementary Table 1) thereby increasing the measured thermal conductivity. In other words, the benefits of the nanoscopic voids and multiple interfaces are best realized below the threshold for stabilization of extended percolative networks.

**Figure 9:**
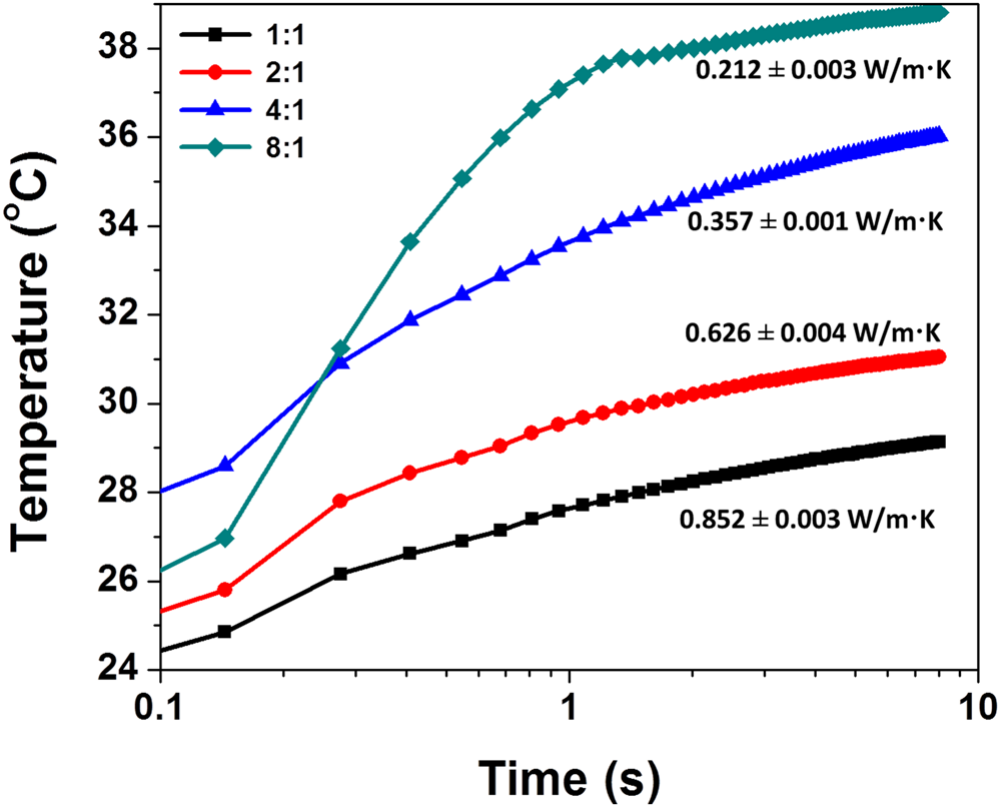
Thermal Conductivity of Nanocomposite Cement Specimens as a Function of HNT:Hydroxyethycellulose Ratio. Plots of the evolution of temperature as a function of time measured using a hot bridge analyzer for unmodified cement and modified cement composites with varying ratios of HNT to hydroxyethylcellulose. The overall loading of hydroxyethylcellulose-modified HNTs is held constant at 2.0 wt.%.

The overall loading of hydroxyethylcellulose-modified HNTs has further been held constant at 2.0 wt.% and the relative ratio of HNT:hydroxyethylcellulose has been varied. The measured thermal profiles are plotted in Fig. 9. The deduced thermal conductivity values decrease with increased HNT:hydroxyethylcellulose loading; a 1:1 sample exhibits a thermal conductivity of 0.852 ± 0.003 W/m.K; a 2:1 sample exhibits a value of 0.626 ± 0.004 W/m.K; a 4:1 sample exhibits a value of 0.357 ± 0.001 W/m.K, and a 8:1 sample yields the lowest thermal conductivity value of this set of samples at 0.212 ± 0.003 W/m.K. The greater volumetric incorporation of nanoscopic voids and the increased number of interfaces likely contribute to the measured decrease of thermal conductivity.

**Figure 10:**
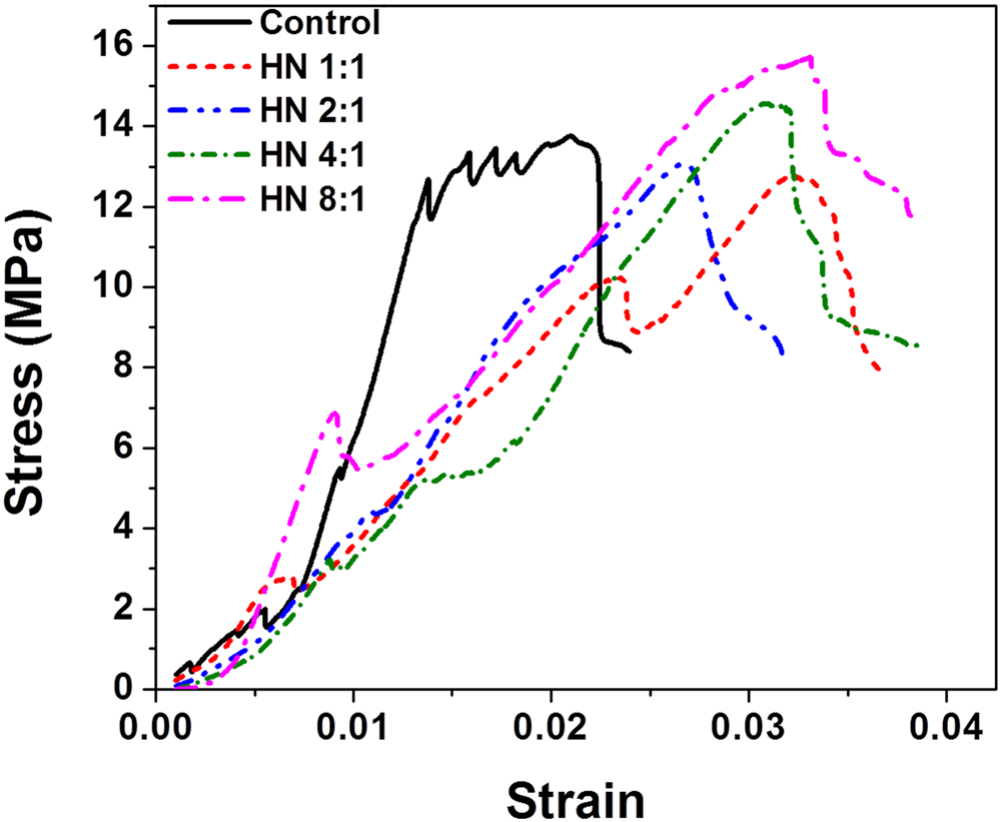
Mechanical Testing of Cement Nanocomposites. Stress *versus* strain curves measured for unmodified and modified cement samples embedded with 2.0 wt.% of hydroxyethylcellulose-functionalized HNTs for different ratios of HNT:hydroxyethylcellulose.

Compressive testing has further been performed for the modified cement samples prepared by varying the HNT:hydroxyethylcellulose ratio while keeping the overall loading of hydroxyethylcellulose-funtionalized HNTs at 2 wt.%. Figure 10 plots the stress *versus* strain plots. The unmodified cement sample has a compressive strength of 13.75 MPa, which given the short drying time (7 days) and size of the sample (5.1 cm diameter, 6.1 cm height) is within the range of values measured for thermal cements. For the samples incorporating hydroxyethylcellulose-modified HNTs, the 1:1 sample has a compressive strength of 12.81 MPa, the 2:1 sample has a strength of 13.08 MPa, the 4:1 sample has a compressive strength of 14.56 MPa, and the 8:1 sample has a compressive strength 15.71 MPa. Supplementary Table 3 summarizes ultimate compressive strength, Young’s modulus, tensile strength, and flexural strength of the samples incorporating hydroxyethylcellulose-modified HNTs. Young’s modulus is directly derived from the stress-strain curve whereas the tensile strength, and flexural strength are estimated according to American Concrete Institute (ACI) methods^41,42^ as detailed in the experimental section. The observed compressive strength values attests that even with incorporation of polymer functionalized HNTs within the matrix, the compressive strength is not deleteriously modified thereby yielding a sample with substantially reduced thermal conductivity that retains the compressive strength of the material. The Young’s modulus is slightly increased for the 1:1–4:1 hydroxyethylcellulose-HNT-modified samples (436.6–445.7 MPa) as compared to the unmodified control (363.5 MPa) implying a small increase in stiffness. The tensile and flexural strengths of concrete are only slightly modified upon fiber reinforcement with higher values observed at higher HNT loadings (Supplementary Table 3). In general, the mechanical properties of hydroxyethylcellulose-modified HNT reinforced concrete upon incorporation of 2 wt.% of hydroxyethylcellulose-modified HNTs in the hydroxyethylcellulose:HNT range of 1:1–4:1 are not substantially altered from unmodified cement. However, further increasing the loading of HNTs (e.g., to a hydroxyethylcellulose:HNT ratio of 8:1) brings about an increase in the overall mechanical strength as well as stiffness. The incorporation of nanoscopic enclosed voids that can increase phonon boundary scattering without weakening the host matrix underpins the observed desirable combination of thermal and mechanical properties.

## Conclusions

The available palette of thermal cements is rather sparse despite the urgent need for low-thermal-conductivity cement for applications such as SAGD wherein heat loss to the external environment represents a considerable expense. The inclusion of additives to form nanocomposites to reduce thermal conductivity without deleteriously impacting load-bearing ability has been examined across a multidimensional compositional space (type of fiber and polymer, loading, ratio of filler/polymer). The inclusion of polymer-modified fillers has been examined for three distinct fibrous additives: CNTs, HNTs, and jute fibers; and three distinct polymers hydroxyethylcellulose, PVA, and poly(acrylic acid). Hydroxyethylcellulose-modified fibers can be embedded within cement without discernible microannulus formation and exhibit a porous microstructure. CNTs bring about an enhancement of thermal conductivity, whereas jute fibers and HNTs substantially reduce thermal conductivity. The diminution of thermal conductivity is most pronounced for HNTs as a result of the nanoscale voids and the phonon boundary scattering at multiple interfaces. The thermal conductivity appears to be lowest below the percolation threshold. Modified cement nanocomposites incorporating HNTs along with hydroxyethylcellulose in a 8:1 ratio with an overall loading of 2 wt.% exhibit the lowest measured thermal conductivity of 0.212 ± 0.003 W/m.K, which is substantially reduced from the thermal conductivity of unmodified cement (1.252 W/m.K). The mechanical properties of the hydroxyethylcellulose-HNT nanocomposite cements are not deleteriously impacted upon incorporation of hydroxyethylcellulose-modified cement. The development of novel nanocomposite cements with reduced thermal conductivity fulfills a substantial need for well cementing in the SAGD process.

## Materials and Methods

Portland Class G cements employed in this study were sourced from Cenovus Energy, Inc. The classification of cement cited here is as per the norms established by the American Petroleum Institute (Class A—H)^43^. The choice of this cement stems from its ubiquity in drilling and completion operations in the Canadian Oil Sands.

CaCl_2_ (Sanjel), SiO_2_ flour (Sanjel), Multi-walled CNTs (CheapTubes), HNT nanoclays (Aldrich), twisted jute twine (SecureLine), hydroxyethylcellulose (Natrosol^TM^ 250H4Br PA; Ashland Chemicals), polyacrylic acid (Carbopol^TM^; Acros Organics), poly(vinyl alcohol) (PVA; Aldrich), poly(acrylic acid) (Acrylsol^TM^; Dow Chemicals), were obtained and used without additional purification.

### Preparation of colloidal dispersion of polymer-functionalized fillers

The polymer-functionalized fillers were obtained by mechanically mixing fibrous fillers and the polymer in water. Several types of fibrous fillers were examined for their ability to facilitate modulation of the thermal properties of the cement: (a) CNTs (b) HNTs, and (c) cellulosic jute fibers. Several polymers were further investigated that can grafted to the cement matrix: (a) hydroxyethylcellulose (Natrosol^TM^); (b) polyacrylic acid (Carbopol^TM^), (c) PVA, and (d) poly(acrylic acid) (Acrysol^TM^). Initially, 2 g of the fibrous filler was placed within a plastic container and next a dispersion comprising 0.5 g of polymer blended in 10 mL of water was added. The obtained dispersion was ultrasonicated for 10 min using a bath sonicator to ensure homogenization of the mixture. The relative mass ratios of the filler and polymer was varied from 8:1 to 1:1 while keeping the total mass at 2.5 g.

### Preparation of cement nanocomposites incorporating polymer-functionalized fillers

Unmodified cement specimens were constituted by mixing and drying the cementitious slurry, which was prepared by mechanically stirring 223.62 g of G-class cement, 89.58 g of silica flour, and 6.26 g of CaCl_2_ in 133 mL of de-ionized water for 10 min. The well-mixed cementitious slurry was immediately transferred to a plastic mold with a diameter of 52 cm and height of 104 cm and cured for 7 days. The preparation of modified cement nanocomposites followed exactly the same procedure with the addition of pre-prepared polymer-functionalized fillers during the mixing step. After drying for 7 days, the cement slurry was extracted from the mold for further characterization. The obtained cement slab typically had a diameter of 50 cm and a height of 100 cm. Cement specimens without addition of CaCl_2_ were also prepared following the same procedure as described above except addition of CaCl_2_.

### Structural and Morphological Characterization

The morphological characteristics of the cement samples, with and without inclusion of polymer-enrobed fillers, were evaluated using a Leica EZ4 stereomicroscope equipped with a KL 1500 LCD detector and a FEI Quanta 600 field emission scanning electron microscope (FE-SEM) equipped with a conventional Everhart-Thornley detector, back-scattered electron detector, and IR-CCD chamber camera operated at an accelerating voltage of 10–20 kV. The chemical composition of cement samples was analyzed by EDS using an Oxford Instruments silicon drift detector. The samples for SEM/EDS characterization were prepared by fracturing the cured large cement slab with a mechanical clamp; ultra-thin sections with a smooth surface were selected, which were adhered to the SEM sample holder with carbon tape.

High-resolution transmission electron microscopy (TEM) images were acquired using a FEI-Tecnai G2 F20 ST instrument at an accelerating voltage of 200 kV. The samples for TEM characterization were prepared by mechanically fracturing with a mechanical clamp and grinding by mortar and pestle to obtain a powered cement sample, which was further dispersed in ethanol. Next, a few drops of the dispersion were transferred to a carbon-supported Cu TEM grid.

Fourier transform infrared (FT-IR) spectra were obtained using a Bruker VERTEX 70 instrument in the range of 4000—500 cm^−1^ with a spectral resolution of 4 cm^−1^.

Powder X-ray diffraction (XRD) measurements were performed using a Bruker D8-Focus Bragg-Brentano X-ray Powder Diffractometer with a Cu Kα radiation source (λ = 1.5418 Å) in the 2*ϑ* range from 10–60°.

### Thermal Conductivity Measurements

Thermal conductivity measurements were performed with a hot point sensor on a Linseis Transient Hot Bridge (THB) 100 – Thermal Conductivity Meter. The sample setup was performed by placing one cylinder flat on a table; next, the hot point sensor was placed at a desired measuring spot, and finally a second cylinder of the same exact composition was placed directly atop the first cylinder, thereby sandwiching the sensor and minimizing exposure to the open air. The measurements were performed using 10 mA of current and 40 mW of power over a period of 8 s and were performed in triplicate for each configuration with a wait time of 100 s in between measurements.

### Compressibility Testing

The compressive strength of the cement nanocomposite specimens were measured on an Instron 5982 Mechanical Testing Device using a servo-controlled 100 kN load capacity at a displacement rate of 5 mm/min. The dimensions of the cement specimens measured 5.1 cm in diameter and 6.1 cm in height.

### Calculation of Young’s Modulus, Estimated Tensile Strength, and Estimated Flexural strength

Young’s modulus (elastic modulus) is directly derived from stress-strain curve by measuring the slope in the linear region from the initial loading to the yield point. The tensile strength and flexural strength were estimated in accordance with the American Concrete Institute (ACI) procedures as per^41,42^:

1 $${{f}}_{{t}}=0.56\sqrt{{{f}}_{{c}}}\cdots $$

2 $${{f}}_{{r}}=0.62\sqrt{{{f}}_{{c}}}\cdots $$

where *f*_*c*_ is the compressive strength, *f*_*t*_ is the splitting tensile strength, and *f*_*r*_ is the flexural strength.

## Electronic supplementary material

Supporting Information
