# Supplementary material for: Incorporation of Hydroxyethylcellulose-Functionalized Halloysite as a Means of Decreasing the Thermal Conductivity of Oilwell Cement
Source: Sci Rep. 2018 Nov 1;8:16149. doi: 10.1038/s41598-018-34283-0 (PMC6212445; doi:10.1038/s41598-018-34283-0)
Supplement: Supplementary file 1 — Supporting Information [file 41598_2018_34283_MOESM1_ESM.docx]

**Incorporation of Hydroxyethylcellulose -Functionalized Halloysite as a Means of Decreasing the Thermal Conductivity of Oilwell Cement**

Junsang Cho, ^1,2‡^Gregory R. Waetzig, ^1,2‡^ Malsha Udayakantha,^1,2^ Claire Y. Hong,^3^ and Sarbajit Banerjee^1,2*^

*‡* These authors contributed equally to this work.

^1^Department of Chemistry, Texas A&M University, College Station TX 77843-3255

^2^Department of Materials Science & Engineering, Texas A&M University, College Station TX 77843; *E-mail: banerjee@chem.tamu.edu

^3^Cenovus Energy, Inc., 500 Centre St. S., Calgary, AB T2P 0M5, Canada

Corresponding author: S.B.: banerjee@chem.tamu.edu

**
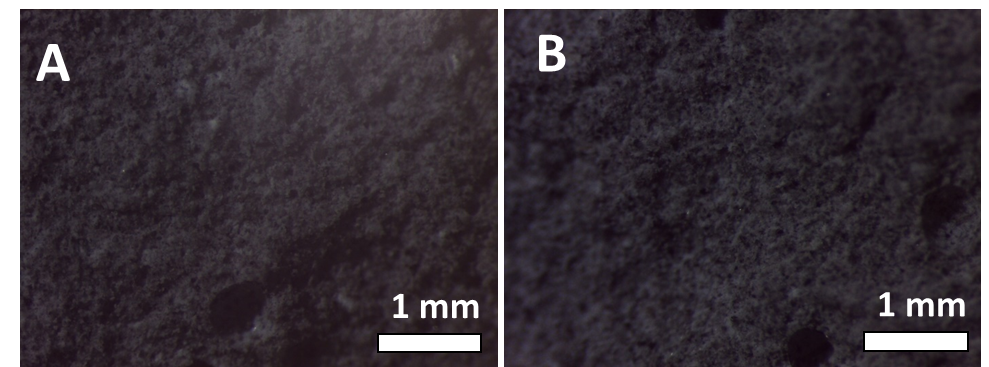
**

**Supplementary Figure 1. Stereomicroscopy Characterization of Modified Cement Nanocoposites.** Stereomicroscopy images of modified cement nanocomposites incorporating a 2 wt.% loading of 2 wt.% of 4:1 polymer—fibrous additive ratio inclusions: (A) HNTs suspended in poly(acrylic acid) and (B) jute fibers suspended in poly(acrylic acid). Large voids spanning several hundred microns are discernible in both panels.

**
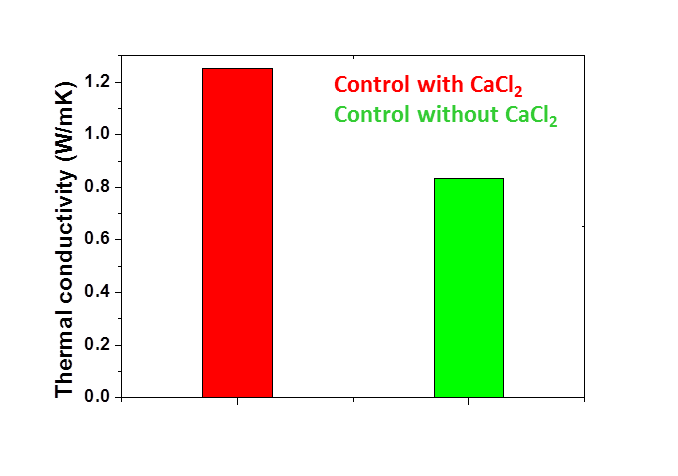

Supplementary Figure 2. Thermal conductivity of cement samples prepared with and without addition of CaCl_2_.** A comparison of thermal conductivity measured using the transient hot bridge method for unmodified cement with and without 2 wt.% of CaCl_2_.

**
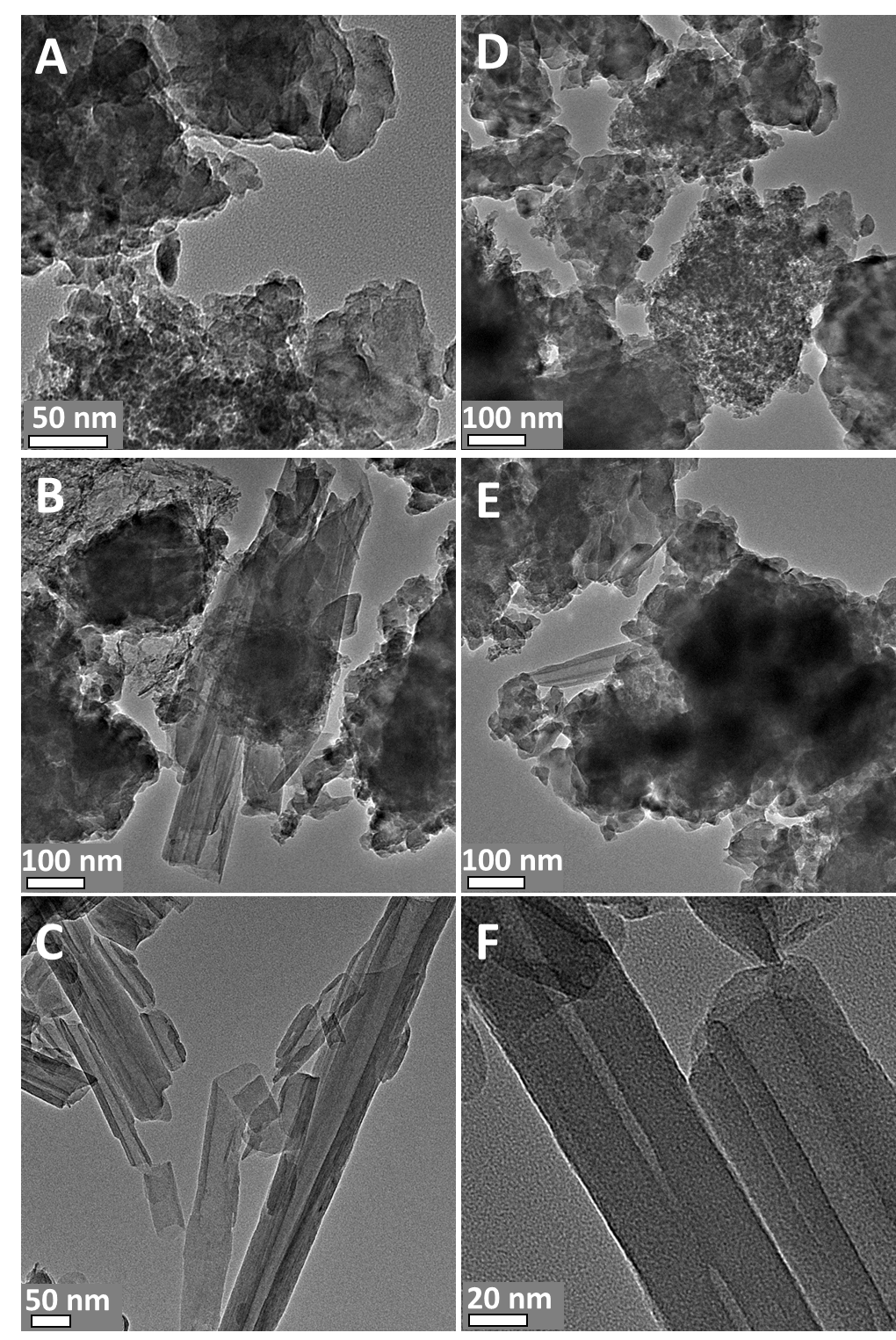
**

**Supplementary Figure 3. TEM Images of Cement Nanocomposites Incorporating Hydroxyethylcellulose-Modified HNTs.** TEM images of A,B) unmodified cement and C,D) modified cement composites incorporating 2 wt.% of hydroxyethylcellulose-modified HNTs. E,F) TEM images of the HNT precursors.

**
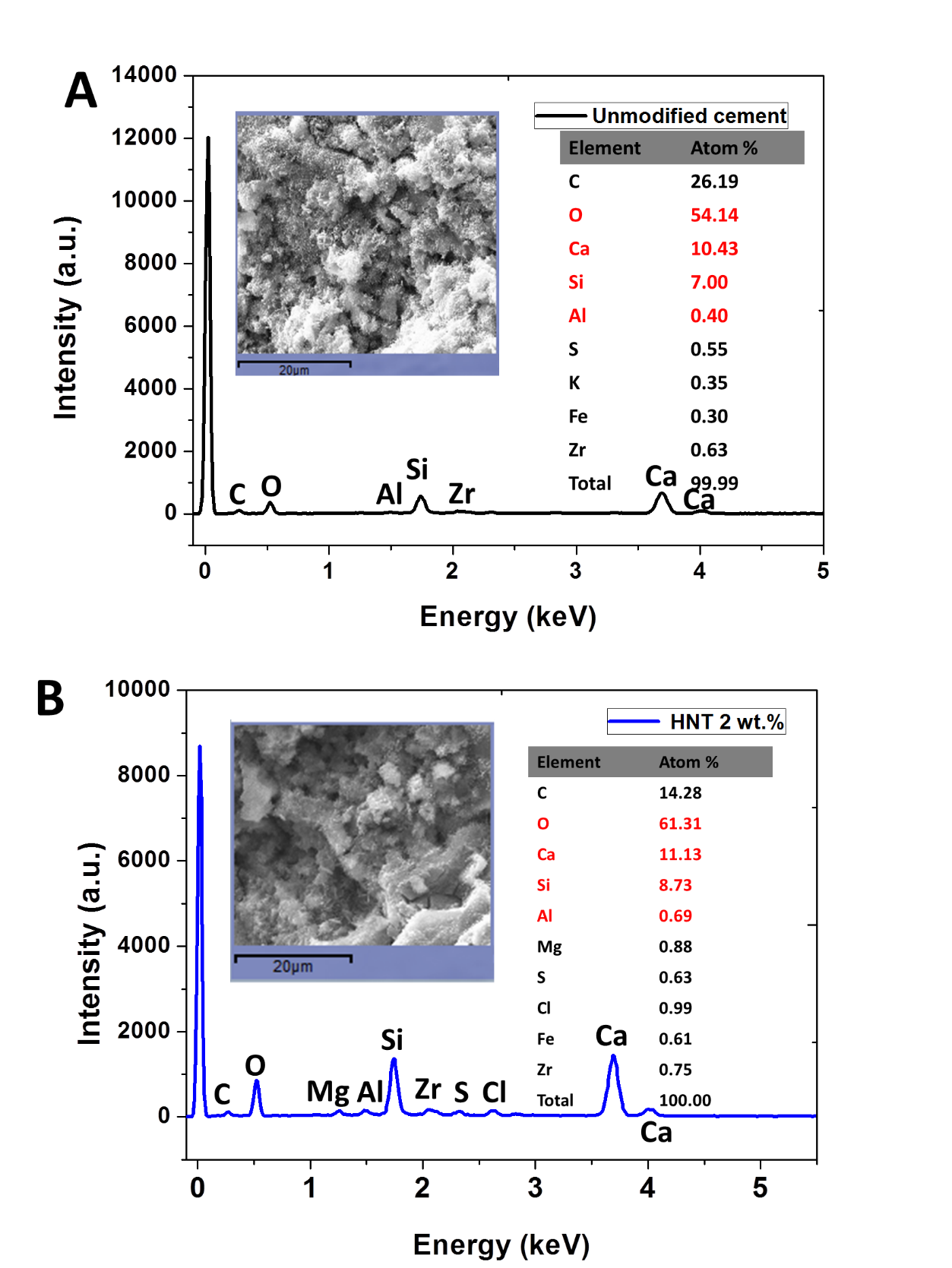
**

**Supplementary Figure 4. Elemental Analysis of Pristine Cement and Modified Cement Composites.** EDS spectra of (A) an unmodified cement specimen and (B) a modified cement composite incorporating 2 wt.% of hydroxyethylcellulose-modified HNTs. The inset showed the SEM images of the samples measured; the table provided alongside lists the elemental compositions for each of the samples.

**
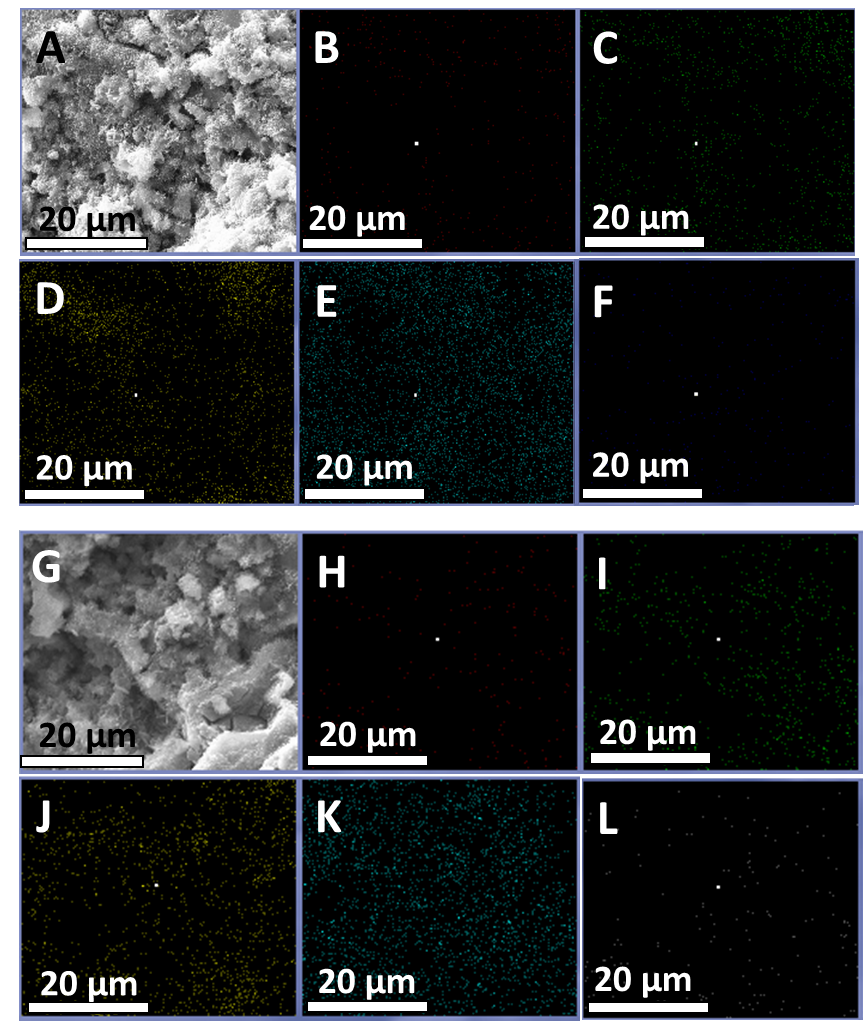
**

**Supplementary Figure 5. Homogenous Distribution of Elements in Modified Cement Nanocomposites.** EDS elemental maps measured for A-F) unmodified cement and G-L) modified cement composite incorporating 2 wt.% of hydroxyethylcellulose-modified HNT: (A,G) SEM images, (B,H) carbon elemental maps (red); (C,I) O elemental maps (green); (D,J) Si elemental maps (yellow); (E,K) Ca elemental maps (cyan); (F,L) K elemental maps (grey).

**
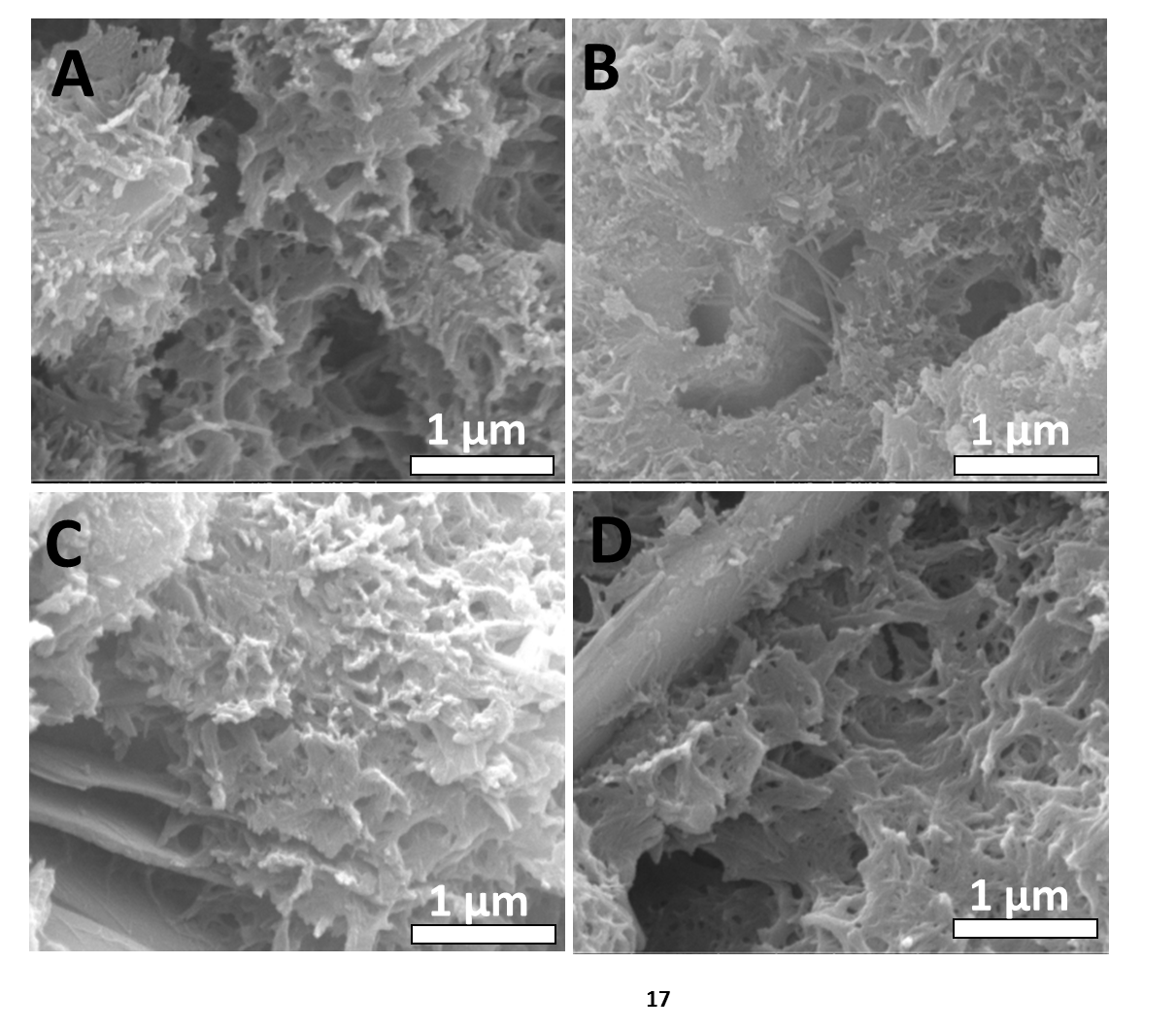
**

**Supplementary Figure 6. Microstructure Formation within Modified Cement Composites.** SEM images of modified cement composites with varying loadings of hydroxyethylcellulose-modified HNTs at a fixed 4:1 stoichiometric ratio of HNTs: hydroxyethylcellulose: (A) 0.5 wt.%; (B) 1 wt.%; (C) 2 wt.%; and (D) 5 wt.%.

| **Fillers** | **Density** | **Diameter** | **Length** | **Aspect ratio** | **Number density (cm^-3^)** |
| --- | --- | --- | --- | --- | --- |
| HNTs | 2.5 g/cm^3^ | 40—50 nm | 300—800 nm | 1:6—1:20 | 3.7×10^12^ |
| CNTs | 1.6 g/cm^3^ | 20—30 nm | 2—3 μm | 1:666—1:1500 | 4.1×10^12^ |
| Jute fibers | 1.5 g/cm^3^ | 10—20 μm | 0.8—1.5 mm | 1:40—1:150 | 2.6×10^5^ |

**Supplementary Table 1**. Density, dimensions, aspect ratio, and number density of additive fillers (HNTs, CNTs, and jute fibers).

| **Thermal conductivity (W/m·K)** | **HNTs** | **CNTs** | **Jute fibers** |
| --- | --- | --- | --- |
| \| Hydroxyethylcellulose \| \| --- \| | 0.424 ± 0.007 | 0.703 ± 0.008 | 1.365 ± 0.015 |
| \| Polyacrylic acid (Carbopol^TM^) \| \| --- \| | 0.853 ± 0.008 | 1.292 ± 0.013 | 0.588 ± 0.006 |
| \| Poly(vinyl alcohol) (PVA) \| \| --- \| | 0.902 ± 0.009 | - | 1.031 ± 0.010 |
| Poly(acrylic acid) (Acrylsol^TM^), | 0.647 ± 0.007 | 0.967 ± 0.010 | 0.940 ± 0.009 |

**Supplementary Table 2.** Matrix corresponding to measurements of thermal conductivity in cement nanocomposites incorporating three different types of fibrous fillers and four distinct polymers at a fixed loading of 2 wt.% with a 4:1 weight ratio of filler/polymer.

| **Sample** | **Ultimate compressive strength (MPa)** | **Young’s modulus (MPa)** | **Estimated tensile strength (MPa)** | **Estimated flexural strength (MPa)** |
| --- | --- | --- | --- | --- |
| Control | 13.75 | 363.5 | 2.08 | 2.30 |
| 1:1 | 12.81 | 445.7 | 2.00 | 2.22 |
| 2:1 | 13.08 | 440.2 | 2.02 | 2.24 |
| 4:1 | 14.56 | 436.6 | 2.14 | 2.36 |
| 8:1 | 15.71 | 976.3 | 2.22 | 2.46 |

**Supplementary Table 3.** Ultimate compressive strength (MPa), Young’s modulus (MPa), estimated tensile strength (MPa), and estimated flexural strength (MPa) with varying ratios of HNT to hydroxyethylcellulose. The overall loading of hydroxyethylcellulose-modified HNTs is held constant at 2.0 wt.%.
